# Deep brain stimulation in the ALIC-BNST region targeting the bed nucleus of stria terminalis in patients with obsessive–compulsive disorder: effects on cognition after 12 months

**DOI:** 10.1007/s00701-022-05351-2

**Published:** 2022-09-02

**Authors:** Johanna Philipson, Matilda Naesstrom, Johannes D. Johansson, Marwan Hariz, Patric Blomstedt, Marjan Jahanshahi

**Affiliations:** 1 Department of Clinical Sciences, Neuroscience, Umeå University, 901 85 Umeå, Sweden; 2 Department of Clinical Sciences, Division of Psychiatry, Umeå University, Umeå, Sweden; 3 Department of Biomedical Engineering, Linköping University, Linköping, Sweden; 4 Unit of Functional Neurosurgery, Department of Clinical and Movement Neuroscience, UCL Queen Square Institute of Neurology, 33 Queen Square, London, UK

**Keywords:** Bed nucleus of stria terminalis, BNST, Obsessive–compulsive disorder, OCD, Deep brain stimulation, DBS, Cognition

## Abstract

**Purpose:**

The aim of this study was to evaluate cognitive effects 12 months after Deep Brain Stimulation (DBS) of the Bed Nucleus of Stria Terminalis (BNST) in patients with refractory Obsessive–Compulsive Disorder (OCD).

**Methods:**

Eight patients (5 female; mean ± SD age 36 ± 15) with OCD were included. A neuropsychological test battery covering verbal and spatial episodic memory, executive function, and attention was administered preoperatively and 12 months after surgery. Medical records were used as a source for descriptive data to probe for any changes not covered by standardized checklists and the Yale-Brown Obsessive Compulsive Scale (Y-BOCS), the primary outcome measure.

**Results:**

At 12 months, seven patients showed response to DBS: three were full responders (i.e., Y-BOCS ≥ 35% improvement), and four were partial responders (Y-BOCS 25–34% improvement). Relative to baseline, there was a slight decline on visuo-spatial learning (p = 0.027), and improved performance on the Color-Word Interference inhibition/switching subtest (p = 0.041), suggesting improvement in cognitive flexibility.

**Conclusions:**

DBS in the BNST for treatment refractory OCD generates very few adverse cognitive effects and improves cognitive flexibility after 12 months of stimulation. The improvement in Y-BOCS and the absence of major cognitive side effects support the BNST as a potential target for DBS in severe OCD.

## Introduction

Obsessive–Compulsive Disorder (OCD) is a severe mental health disorder with an estimated lifetime prevalence of approximately 1–2% [43]. OCD is characterized by anxiety-driven, recurrent, and persistent thoughts that lead to repetitive behaviors or rituals. These obsessions and their associated compulsive behaviors are time-consuming, with sufferers spending at least 1 h, and in more severe cases, the majority of the day, engaging in them [6]. This makes the disorder a significant mental health problem with a pronounced negative impact on quality of life, and an economic burden on society, since many sufferers are unable to work [30, 43, 46].

The etiology and pathophysiology of OCD are not entirely understood. At present, dysfunction in cortico–striato–thalamo–cortical (CSTC) circuits is proposed and is central to most neurobiological models of OCD [41]. In addition, there seems to be a consensus regarding the involvement of the orbitofrontal cortex and the caudate [19, 33, 34, 48]. A meta-analysis focusing on neurocognitive aspects of OCD suggested that the condition is partly a consequence of dysfunctional circuits for response inhibition, cognitive flexibility, planning, goal-directed behavior, working memory, and error monitoring [2].

Psychological treatment, mainly cognitive behavioral therapy (CBT), and pharmacological treatment with selective serotonin reuptake inhibitors (SSRI), selective norepinephrine reuptake inhibitors (SNRI), or in some cases benzodiazepines are the primary treatment options. However, a treatment-refractory subgroup remains. It has been estimated that around 10% of adult OCD patients remain unresponsive to all conventional therapeutic options and hence suffer from symptoms leading to severe functional impairment [17].

Deep Brain Stimulation (DBS) has been suggested as a treatment option for refractory OCD, in carefully selected patients. The first series of OCD patients treated with DBS was published in 1999, with the anterior limb of the internal capsule as the target [39]. Subsequently, areas such as the ventral striatum, including the nucleus accumbens, the limbic part of the subthalamic nucleus (STN), and the bed nucleus of the stria terminalis (BNST), have been used as targets in DBS for OCD [4].

Even though the tolerability and efficacy of DBS have been shown in a growing number of studies, the total number of OCD patients treated with DBS worldwide is just around 250, and the average sample size of all studies has been reported to be five [18]. The cognitive effects of DBS in patients with OCD has been investigated even less (see Table 4) [1, 7, 8, 12, 21, 22, 24, 26, 29, 31, 32, 40, 47]. Generally, diverse brain targets have been used across studies and sample sizes were also limited, ranging between one and 24 patients. The targets for DBS included the ventral striatum-ventral capsule (VS-VC), the nucleus accumbens, the inferior thalamic peduncle, the bed nucleus of the stria terminalis, the subthalamic nucleus, and some combinations of the above. A common denominator to these reports is that DBS, regardless of the target, had no or minor negative influence on performance of the cognitive tests assessed. In fact, various tests showed cognitive improvement, especially concerning attention, memory, and cognitive flexibility.

The safety and potentially superior efficacy of DBS in the BNST, compared to other brain targets, have been reported by Luyten et al. [29]. Cognitive assessments were performed at a short-term follow-up of 3–6 months. Here, we aim to evaluate more long-term cognitive effects 12 months after DBS of the BNST in patients with severe refractory OCD.

## Materials and methods

### Inclusion criteria

Patients fulfilled the following inclusion criteria: age between 18 and 65 years; diagnosis of severe OCD according to DSM-IV criteria; Y-BOCS score ≥ 25; disease duration of ≥ 5 years; disabling symptoms despite adequate trials with at least three different serotonergic acting antidepressants; and augmentation with antipsychotics and CBT. In addition, all patients had to be able to understand the aims of the surgical procedure and consequences of participation in the study, and the ability to provide written informed consent. One patient (P7) did not manage to undergo CBT due to severely disabling OCD. Exclusion criteria included severe psychiatric co-morbidity, current or recent substance abuse, severe self-injurious behavior, pregnancy, and typical surgical exclusion criteria. The study was approved by the local ethical committee, and patients provided their informed consent.

### Patients and study design

Eleven consecutive patients were included in a previous study on DBS in the BNST focusing on the efficacy of the treatment and reported elsewhere [37]. Three patients included in that study could not complete the preoperative cognitive assessment due to their severely disabling mental state. Thus, eight patients (5 female) were included in the present study. Their mean ± SD age at surgery was 36 ± 15 years, with an average of 12 ± 2 years of education. The patients’ demographics, clinical data, and medications are presented in Table 1.

**Table 1:** Demographic, medications, and clinical data

Patient no/sex	Age at onset	Age at surgery	Medication pre	Medication 1YR	Primary diagnosis	Comorbidity
1/F	7	53	Sertraline 300 mg, alprazolam 0.5 mg 1 + 1, zopiclone 7.5 mg	Sertraline 300 mg, alprazolam 0.5 mg 1 + 1	OCD (checking, repetition)	None
2/M	50	59	Citalopram 40 mg	Citalopram 40 mg	OCD (contamination, cleaning)	None
3/F	9	45	Clorprotixen 100 mg, quetiapin 50 mg 1 + 1, lithium 42 mg 3 + 2, zopiklon 7.5 mg, propriomazin 50 mg, phenelzine 15 mg 3 + 3	Clorprotixen 100 mg, quetiapin 50 mg 1 + 1, lithium 42 mg 3 + 2, zopiklon 7.5 mg, propriomazin 50 mg, phenelzine 15 mg 3 + 3	OCD (sexual, aggression)	Bipolar type II
4/M	15	27	Pregabalin 150 mg 1 + 1, quetiapine 600 mg, zopiklon 7.5 mg, tranylcypromine 10 mg 4 + 4, ritalin 20 mg 1 + 1 + 1	Pregabalin 150 mg 1 + 1, quetiapine 600 mg, zolpidem 10 mg, tranylcypromine 10 mg 4 + 4, medikinet 60 mg, melatonin 2 mg	OCD (contamination, cleaning)	Asperger syndrome, ADHD
5 /F	5	21	Escitalopram 20 mg, concerta 36 mg × 2	Escitalopram 20 mg, concerta 36 mg × 2	OCD (contamination, cleaning)	ADHD
6/F	15	22	Aripiprazole 5 mg 1 + 1, amitryptilin 10 mg, fluoxetin 60 mg, alimemazine 40 mg/ml 1.5 ml	Aripiprazole 5 mg 1 + 1, amitryptilin 10 mg, fluoxetin 60 mg, alimemazine 40 mg/ml 1.5 ml	OCD (repetitions)	Anorexia nervosa
7/F	10	27	Olanzapine 10 mg 1 + 1 + 1, zopiklone 5 mg, sertraline 200 mg, mirtazapine 45 mg, alimemazine 60 mg, prometazin 50 mg	Olanzapine 10 mg 1 + 1 + 1, zopiklone 5 mg, sertraline 200 mg, mirtazapine 45 mg, alimemazine 60 mg, prometazin 50 mg	OCD (contamination, cleaning, aggression)	Atypical autism
8/M	7	38	Olanzapine 2.5 mg 2 + 2, zopiklon 7.5 mg, citalopram 60 mg	Olanzapine 2.5 mg 2 + 2, zopiklon 7.5 mg, citalopram 60 mg	OCD (sexual, aggression)	None

### Surgical procedure

**Fig. 1:**
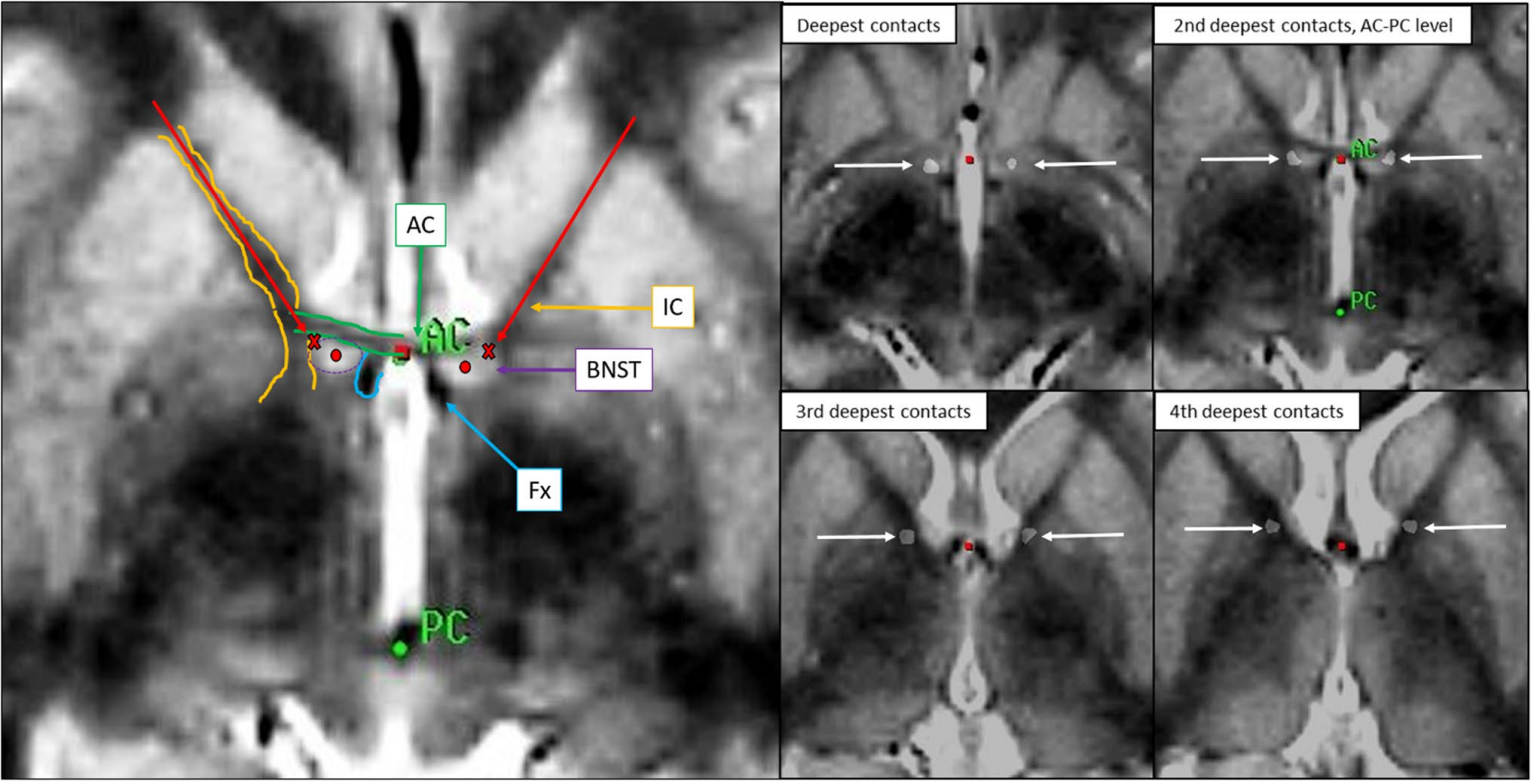
Left: The target is identified at the AC-PC level at the upper lateral border of the BNST (red cross). When advanced 3 mm deeper, the deepest contact will end up in the center of the BNST (red dot). The trajectory is further planned so that it will intubate the internal capsule (red arrow), leaving the two uppermost contacts of the lead in this structure. Right: The actual location of the four electrode contacts in one patient (white arrows). Abbreviations: AC: anterior commissure, PC: posterior commissure, Fx: fornix, BNST: bed nucleus of stria terminalis, IC: internal capsule

**Fig. 2:**
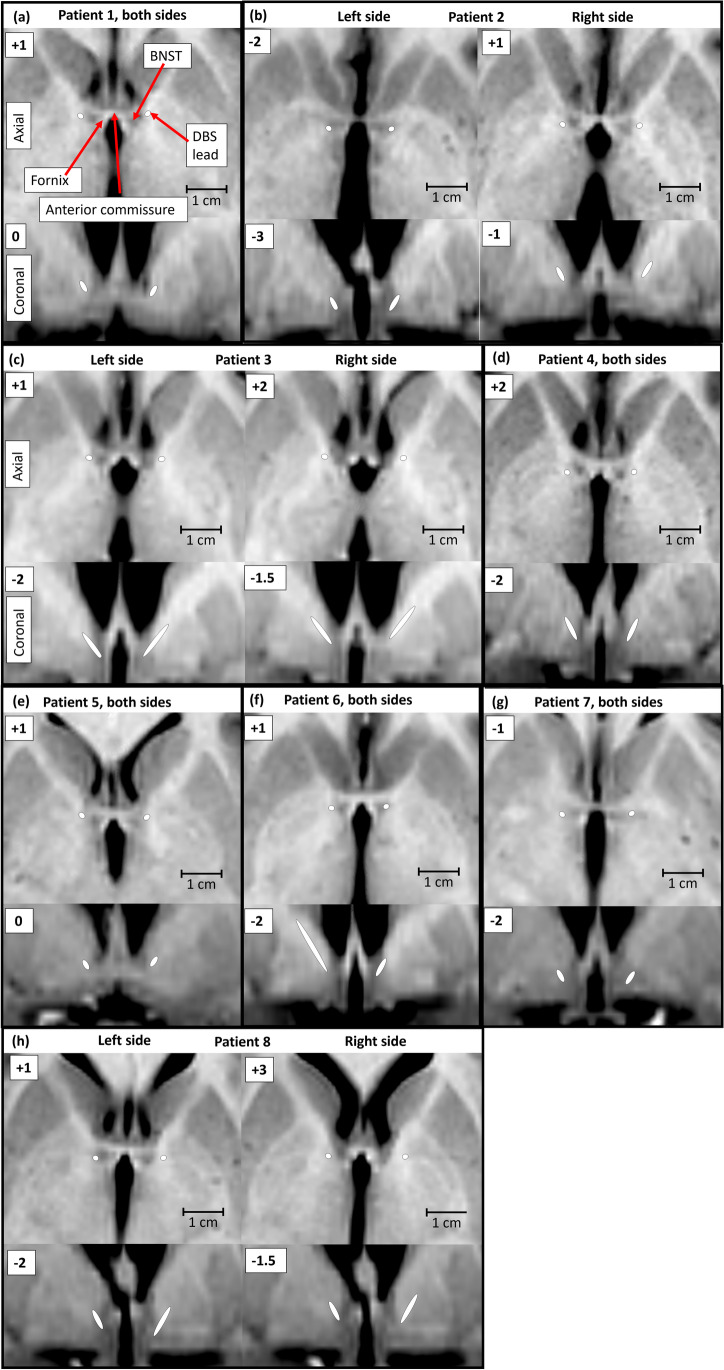
Location of electrodes in the horizontal and coronal plane, centered at the active contact used for stimulation in each individual patient

Surgery was performed under general anesthesia using the Leksell stereotactic frame and a stereotactic proton density-weighted 1.5 Tesla MRI scan enabling individual visualization of the BNST and neighboring structures. Quadripolar electrodes (model 3387, Medtronic, Minnneapolis, MN, USA) were implanted bilaterally and connected to an implantable pulse generator (PC, Medtronic, Minnneapolis, MN, USA). A stereotactic CT scan was performed after surgery and fused with the preoperative MRI scan to identify the electrode position [11]. Figure 1 shows the preoperative targeting and trajectory, and the position of each of the electrode contacts in one of the patients. Figure 2 shows the location of electrodes in the horizontal and coronal plane, centered at the active contact used for stimulation in each individual patient. Table 2 describes in detail the position of active electrodes, stimulation settings, and structures reached by the field of stimulation in each of the eight patients. See Naesström et al. [38] for a more thorough presentation.

**Table 2:** Position of active electrodes at 12 months, stimulation settings, and structures reached by the field of stimulation

Patient	Side	Stimulation settings Contacts*/V/µs/Hz	Location of active contacts	Structures reached by the field of stimulation**
1	Left	1.2/5 V/120 µs/130 Hz	ALIC, BNST	ALIC, GIC, AC, NC, Fx, BNST, GPe, Gpi
	Right	9.10/5 V/120 µs/130 Hz	ALIC, BNST	ALIC, GIC, AC, NC, Fx, BNST, GPe, Gpi
2	Left	1.2/4.2 V/150 µs/130 Hz	ALIC, BNST	ALIC, GIC, AC, NC, Fx, BNST, GPe, Gpi
	Right	9.10/4.2 V/150 µs/130 Hz	ALIC, BNST	ALIC, GIC, AC, NC, Fx, BNST, GPe, Gpi
3	Left	1.2/4.1 V/90 µs/130 Hz	ALIC, BNST	ALIC, GIC, AC, NC, Fx, BNST, Gpi
	Right	9.10/4.1 V/90 µs/130 Hz	ALIC, BNST	ALIC, GIC, AC, NC, Fx, BNST, GPe, Gpi
4	Left	1.2/4.9 V/90 µs/130 Hz	ALIC, BNST	ALIC, GIC, AC, NC, Fx, BNST, GPe, Gpi
	Right	9.10/4.9 V/90 µs/130 Hz	ALIC, BNST	ALIC, GIC, AC, Fx, BNST, GPe, Gpi
5	Left	1.2/3.8 V/60 µs/130 Hz	ALIC, BNST	ALIC, GIC, AC, NC, Fx, BNST, GPe, Gpi
	Right	9.10/3.8 V/60 µs/130 Hz	ALIC, BNST	ALIC, GIC, AC, NC, Fx, BNST, GPe, Gpi
6	Left	1.2/4.4 V/60 µs/130 Hz	ALIC, BNST	ALIC, GIC, AC, NC, Fx, BNST, GPe
	Right	9.10/4.4 V/60 µs/130 Hz	ALIC, BNST	ALIC, GIC, AC, NC, Fx, BNST, GPe, Gpi
7	Left	1.2/4.3 V/90 µs/130 Hz	ALIC, BNST	ALIC, GIC, AC, NC, Fx, BNST, GPe, Gpi
	Right	9.10/4.3 V/90 µs/130 Hz	ALIC, BNST	ALIC, GIC, AC, Fx, BNST, GPe, Gpi
8	Left	1.2/3.5 V/90 µs/130 Hz	ALIC	ALIC, GIC, AC, NC, BNST, GPe, GPi
	Right	9.10/3.5 V/90 µs/130 Hz	ALIC, BNST	ALIC, GIC, AC, NC, Fx, BNST, GPe, Gpi

^*^Only monopolar settings were used. ** Calculations of the electric field magnitudes corresponding to stimulation of the largest axons in the brain have been performed with finite element method simulations (COMSOL Multiphysics 5.3a, COMSOL, Sweden) using patient-specific tissue properties (see Naesstrom et al.^38^). Abbreviations: *AC* anterior commissure. *ALIC* anterior limb of internal capsule. *BNST* bed nucleus of stria terminalis. *Fx* fornix. *GIC* genu of internal capsule. *GPe* globus pallidus externa. *GPi* globus pallidus interna. *NC* caudate nucleus. *V* volt. *μs* microseconds. *Hz* Hertz

### Clinical evaluation

The clinical evaluation consisted of pre- and post-operative ratings, using the Yale-Brown Obsessive Compulsive Scale (Y-BOCS). The Y-BOCS is a 10-item clinically rated scale consisting of items probing for obsessions and compulsions. The severity of OCD is defined by time spent on obsessions/compulsions, level of anxiety, and functioning caused by OCD symptoms as well as the ability to control obsessions and compulsions. The scale ranges from 0 to 40. Zero to seven points indicate subclinical, 8–15 mild, 16–23 moderate, 24–32 severe, and 33–40 extreme OCD symptoms. Additional descriptive data about patient’s everyday life was also recorded.

### Neuropsychological evaluation

The patients were assessed preoperatively (baseline) and 12 months postoperatively. Alternative versions of tests primarily focusing on memory were used at the postoperative assessment to minimize potential practice effects. A short description of each test is presented below.

#### Non-verbal reasoning and fluid intelligence

Two tests were completed at baseline, as part of the pre-operative screening. The Raven’s Colored Matrices is a non-verbal measure of fluid intelligence and non-verbal reasoning with a maximum score of 36. The results are presented as percentiles, standardized based on age group. The Wechsler Adult Intelligence Scale-IV (WAIS-IV) subtest “Matrices” is also a measure of non-verbal reasoning and fluid intelligence. Raw scores are converted to age-corrected scaled scores, ranging from 1 to 19, with higher scores indicating better performance.

#### Memory

Claeson-Dahls test [13] is a Swedish test of verbal learning and memory. Ten words are read out to the patient and after a 15-s pause, the patient recalls as many as possible. After 30 min, the patient is asked to again recall as many words as possible. In addition to delayed recall, a delayed recognition task is also administered. Four alternate versions are available for retesting. Results are calculated based on the number of words recalled and the number of times the list of words needs to be repeated. A low score indicates a faster learning curve.

Brief Visuospatial Memory Test (BVMT-R) [9] is a test for assessment of visual memory and comprises six alternate, equivalent forms (Forms 1 through 6). Each form consists of 6 geometric figures and 12 recognition items. The figures are presented three times to assess short-term spatial memory and learning. After 30 min, delayed recall is tested followed by a recognition test. Scores are given for accuracy and location/placement of the figures. Scores range from 0 to 12 in each of the three trials, thus a maximum of 36 in the learning task and 12 in the recognition task. Higher scores indicate better visual memory.

Wechsler Adult Intelligence Scale IV (WAIS-IV) [53] and Digit forward and backward are tests of attention and short-term verbal working memory. Digit span backward also engages executive functions. Digit span is measured for forward and reverse-order (backward) recall of digit sequences. Scores range from 0 to 18 forward and 0 to 16 backwards with higher scores indicating better performance. Raw scores are converted to age-corrected scaled scores, ranging from 1 to 19, with higher scores indicating better performance.

#### Executive functions

Delis-Kaplan Executive Function system (D-KEFS) [16]: This is a set of standardized tests assessing higher-level cognitive functions, referred to as executive functions.

Trail Making Test: This is a test of visual attention, behavioral regulation, task switching/cognitive flexibility, inhibition of perseverative responding, and processing speed. The test consists of five individual subtests: visual scanning; number sequencing; letter sequencing; number-letter switching; and motor speed. Scores are based on the time needed to complete the individual subtests. Results are transformed to scaled scores, ranging from 1 to 19, where a higher score indicates better performance.

Verbal fluency: Test of verbal functioning consisting of three tasks: phonemic fluency, semantic fluency, and switching category fluency. The participant’s score in each task is the number of unique correct words, excluding intrusion and repetition errors. The participant is asked to shift between two semantic categories in the switching category task. Raw scores are transformed into scaled scores ranging from 1 to 19, where a higher score indicates better performance.

Color Word Interference test (CWIT): measures aspects of frontal lobe-related executive functions, such as processing speed, cognitive flexibility and impulse control, and the ability to suppress a habitual response. The CWIT comprises four subtests: color naming; word reading; color word interference/inhibition; and inhibition-switching*.* The total time to complete each subtest and the number of self-corrected and uncorrected errors are recorded. Scores are transformed into scaled scores ranging from 1 to 19, where a higher score indicates a better result.

#### Attention

Dichotic listening task [25] is a test for studying brain asymmetry in auditory processing. The patient is presented stereophonically with two different auditory stimuli, i.e., consonant–vowel (CV) syllables simultaneously, one to each ear. Three conditions measure different kinds of cognitive processes: a lateralized perceptual process (non-forced condition), an attention process (forced-right), and an executive cognitive control process (forced-left condition). The non-forced condition is typically used to determine speech lateralization. The results from the forced attention conditions are calculated as a percent correct score of the 30 dichotic stimuli presented to the right and left ear, respectively. The raw score is transformed into a T-score ranging from < 20 to > 80. Interpretations of the T-scores indicate the patient’s ability to shift auditory attention.

Integrated Visual and Auditory Continuous Performance Test (IVA) [42] is a computerized continuous performance test designed to evaluate visual and auditory attention, inhibition, and cognitive control. The participants are instructed that they will see or hear the numbers “1” or “2.” They are then asked to click the mouse when presented with a visual or auditory “1” and inhibit action when presented with a “2.” Results are provided for global attentional functioning and cognitive control, as well as separate scores for visual and auditory attention and response control. Scores are presented as T-scores ranging from < 20 to > 80 with a higher score indicating better performance.

### Statistics

Statistical analyses of neuropsychological data were performed on normative scores corrected for age, sex, and education. Due to the small group size and the data not being normally distributed, the Wilcoxon signed rank test for paired samples was used to compare pre- and postoperative scores. A p-value of ˂ 0.05 was considered significant.

## Results

### Yale-Brown Obsessive–Compulsive Scale (Y-BOCS)

The mean (± SD) Y-BOCS score was 32.5 ± $2.72$ at baseline and 21.63 ± 4.4 at 1 year (p = 0.01). Three patients were classified as responders (i.e., with a ≥ 35% improvement on Y-BOCS) and four patients as partial responders (i.e., with a 25–35% improvement on Y-BOCS). There was one non-responder at the 12-month follow-up. Table 3 shows the Y-BOCS scores of each of the eight patients in this study.

**Table 3:** Pre- and postoperative scores on the Yale-Brown Obsessive–Compulsive scale (Y-BOCS) in each patient

Patient no	Preop Y-BOCS	Post op Y-BOCS	% YBOCS change
1	35	14	60%
2	29	26	10%
3	31	21	32%
4	35	23	34%
5	32	24	25%
6	29	17	41%
7	36	27	25%
8	33	21	36%

### Stimulation parameters

At the12-month follow-up, all patients had monopolar stimulation with identical settings on both electrodes. The mean stimulation parameters were 4.1 V (range 3.2–5), 90 µs (range 60–150), and 130 Hz (± 0). For detailed description of active contacts and parameters used for chronic stimulation as well as exact anatomical location of the active contacts in each individual patient, please see Table 2 and Fig. 2.

### Descriptive data

Descriptive data revealed important changes in the daily lives of the patients. Four of the patients (no. 1–4 in Table 1) had lived in isolation, either in their apartment or in in-patient care for years prior to surgery. Their OCD symptoms prohibited most social interactions. One year after surgery, all four patients could be more socially active both inside and outside the home. This was also the case for patient no. 2 who, although showing only a 10% improvement on the Y-BOCS, i.e., classified as non-responder, was able to socialize to a greater extent 1 year after surgery. His previous intrusive thoughts had bordered on delusional thinking. Once that subsided, he could engage more in social settings, even though the compulsive behaviors were still present. One patient (no. 6) was able to leave in-patient care for the first time in 3 years and instead resides in an out-patient facility independently managing most activities of daily living (ADL). As for the other participants, patient 5 spent less time on compulsive behavior, patient 7 showed a decrease in self-harm and gained the ability to withstand OCD symptoms with help from staff at the out-patient facility, and patient 8 could increase his part-time working hours from 50 to 75% 1 year after surgery.

### Changes in cognition after DBS surgery

The results of the neuropsychological tests of cognition at baseline and 12 months after surgery are presented in Table 4. Estimates of general intelligence were obtained preoperatively either with the Ravens Colored Matrices or The Wechsler Adult Intelligence Scale IV (WAIS-IV) Matrices. Mean scores were within the 95th %-tile on the Ravens Colored Matrices and the mean scaled scores on the WAIS-IV Matrices were 9.17 ± 2.93, which are within the normal range. There were no changes in global cognition or in the major cognitive domains. Relative to baseline, only visuo-spatial learning showed a statistically significant decline of just over 1 SD 12 months after surgery (p = 0.027). The Color-Word Interference inhibition/switching subtest, a test which requires switching between two response sets and hence measures cognitive flexibility, showed improved performance (p = 0.041) 12 months after surgery. None of the changes on the other measures of cognition were significant (all p > 0.05).

**Table 4:** The mean (SD) scores on tests of cognitive function before and 12 months after surgery

Test	Baseline mean (SD)	12 months mean (SD)	p-value Baseline vs 12 months
*Memory*			
Claeson Dahl Verbal Learning (T-score)	47.00 ( 8.12)	52.6 (10.42)	p = 0.233
Claeson Dahl Retention (T-score)	47.75 (10.77)	44.50 (13.15)	p = 0.735
Benton Visual Memory Test-Revised (BVMT-R) Recall (T-score)	37.13 (11.95)	44.13 (13.42)	p = 0.176
BVMT-R Learning (T-score)	58.13 (9.51)	46.75 (3.85)	**p = 0.027**^**# #**^
BVMT-R Delayed recall (T-score)	43.00 (13.13)	46.13 (12.07)	p = 0.483
Digit forward (scaled score)	9.25 (2.96)	9.38 (2.56)	p = 0.777
*Executive Function*			
Phonemic fluency (scaled score)	11.88 (3.36)	11.25 (3.58)	p = 0.096
Semantic fluency (scaled score)	12.63 (2.72)	11.13 (3.14)	p = 0.092
Semantic switching (fluency scaled score)	10.88 (1.96)	11.38 (1.85)	p = 0.380
Correct switches (scaled score)	11.25 (1.91)	11.88 (1.55)	p = 0.222
Color-Word Inhibition Test (CWIT), inhibition—completion time (scaled score)	8.63 (3.07)	9.13 (3.98)	p = 0.394
CWIT, inhibition/switching—completion time (scaled score)	8.00 (3.30)	10.00 (2.45)	**p = 0.041 **^**#**^
CWIT, inhibition errors (scaled score)	11.25 (0.89)	10.25 (3.06)	p = 0.705
CWIT, inhibition/switching errors (scaled score)	10.00 (1.51)	10.88 (0.64)	p = 0.096
Trail Making Test, letter-number switching Completion time (scaled score)	8.25 (1.91)	9.50 (2.73)	p = 0.176
Trail Making Test, letter-number switching total errors (scaled score)	9.88 (2.10)	10.63 (1.77)	p = 0.330
Digit backward (scaled score)	9.50 (2.45)	10.75 (1.75)	p = 0.351
*Attention/working memory*			
Integrated Visual and Auditory Test (IVA) (full scale response auditory)	100.71 (11.12)	89.86 (24.22)	p = 0.204
IVA (full scale response visual)	84.29 (29.44)	91.86 (22.54)	p = 0.236
IVA (full scale attention auditory)	99.29 (11.97)	105.14 (15.93)	p = 0.075
IVA (full scale attention visual)	92.57 (10.15)	100.86 (16.66)	p = 0.176
Dichotic listening Non-forced RE (T-score)	45.88 (12.08)	49.83 (19.23)	p = 0.249
Dichotic listening Non-forced LE (T-score)	49.63 (7.52)	49.33 (11.76)	p = 0.480
Dichotic listening Forced Right RE (T-score)	46.38 (11.80)	44.83 (9.95)	p = 0.752
Dichotic listening Forced Right LE (T-score)	52.25 (11.12)	52.83 (11.39)	p = 0.686
Dichotic listening Forced Left RE (T-score)	51.38 (7.29)	56.83 (8.26)	p = 0.136
Dichotic listening Forced Left LE (T-score)	46.38 (7.35)	44.33 (8.17)	p = 1.000

^#^ Indicating improvement. ## Indicating deterioration. Abbreviations: *RE* right ear, *LE* left ear, T-score = mean 50 (SD ± 10)

## Discussion

In this study on eight patients with severe OCD who had DBS in the BNST, there were virtually no adverse effects on cognition, other than a worsening of visuo-spatial learning 12 months after surgery. Our results are in line with several of the few previously conducted studies of DBS in other brain targets than the BNST listed in Table 5, reporting no, or very few, changes in global cognitive function after DBS in patients with refractory OCD [21, 26, 31].

**Table 5:** Summary of studies focusing on cognitive effects of DBS in patients with OCD

Author	Target	Sample size	Time of assessment	Neuropsychological tests	Main findings
Aouizerate et al., 2004 [7]	Ventral caudate nucleus/nucleus accumbens	1	Baseline, 1, 6 months post op	Free and Cued Selective Reminding Test, Benton Visual Retention Test, Trail Making Test A + B, Wisconsin Card Sorting Test, Stroop, Zazzo’s selective attention test, Isaacs set test	Improvement: verbal and visual memory, cognitive flexibility
Abelson et al., 2005 [1]	Base of internal capsule	4	Baseline, 6 months post-op, on/off	California Verbal Learning Test, Category Test, Finger Tapping Test, Fragmented Pictures, Grooved Pegboard, Money Road Map Test, Visual-Spatial Learning Test, Wechsler Adult Intelligence Scale, Wechsler Memory Scale, Wisconsin Card Sorting Test. ON/OFF: Corsi block span, Digit Span, Stroop	No changes in cognition between on and off setting. Improvement of motor speed and working memory at 6 months
Mallet et al., 2008 [28]	Subthalamic nucleus	16	Baseline, 3, 6, 7, 10 months post op	Hopkins verbal learning test, Trail Making Test, Stroop, Verbal fluency, Wechsler Memory Scale	No changes in cognition reported, on/off stimulation
Jiménez-Ponce et al., 2009 [24]	Inferior thalamic peduncle	5	Baseline, 3, 6, 9, 12 months post-op	Block Design, Wisconsin Card Sorting Test, Finger Tapping Test, Fluency, Stroop, Token Test	No changes in cognition reported
Goodman et al., 2010 [19]	Ventral capsule/ventral striatum	6	Baseline, monthly evaluation 1–12 months post-op	Wisconsin Card Sorting Test, Fluency, Hopkins verbal learning test, Grooved Pegboard, Tower of London, Digit Span	No changes in cognition reported
Huff et al., 2010 [21]	Nucleus accumbens	10	Baseline, 3, 6, 9, 12 months post-op	Phonemic fluency, Tower of London, Continuous Performance Test	Improvements found in attention. No other changes in cognition reported
Grant et al., 2011 [20]	Nucleus accumbens	1	Baseline, 8 months	Stop Signal Task, Cambridge Neuropsychological Test Automated Battery (CANTAB): intra/extra dimensional shift, Cambridge gambling task	No changes in cognition reported
Mantione et al., 2015 [29]	Nucleus accumbens	16 cases, 14 control	Baseline, 3 weeks, 8 months post-op	Dutch National Adult Reading Test, Raven’s Advanced Progressive Matrices, Dutch Rey Auditory Verbal Learning Test, WAIS Digit Span, Rey Complex Figure, Stroop Color-Word, Verbal Fluency, Trail Making Test, Wisconsin Card Sorting Test, Tower of London, Continuous Performance Test (CPT), Identical Pairs, Digit Symbol substitution, Purdue Pegboard	DBS group worse results on Digit Symbol substitution at baseline. At 3 weeks post-op, DBS group showed lower results on Rey Complex Figure “Copy” and on verbal fluency. At 8 months post-op, Rey Complex Figure (copy) still lower than controls, but verbal fluency had improved
Luyten et al., 2015 [26]	Bed nucleus of the stria terminalis (BNST) and anterior limbs of the internal capsule (ALIC)	24	Baseline, and at crossover (3, 6 months)	Complex Figure Test of Rey, Audio Verbal Learning Test, Wisconsin Card Sorting Test, Word Fluency test, Raven Standard progressive matrices, Stroop Color-Word, and Trail Making Tests A and B	Stimulation improved scores on the Complex Figure Test of Rey and subtests of the Auditory Verbal Learning Test, and appeared to yield lowered scores on subtests of the Stroop test, i.e., shorter completion time, proposing less interference, and improved executive functioning
Choudhury et al., 2017 [11]	Anterior limb of internal capsule	1	Baseline, 35 and 51 months post-op	Montreal Cognitive Assessment (MoCA), Wechsler Test of Adult Reading, WAIS Digit Span, Verbal Sustained Attention Test, Symbol digit coding, Symbol Search, Stroop Color-Word, Rey Auditory Verbal Learning Test, Brief Visuospatial Memory Test–Revised, Controlled Oral Word Association Test, Wisconsin Card Sorting Test	MoCA below average at baseline, which improved at both 35 and 51 months post-op. No cognitive declines reported. Improvements across several cognitive domains
Barcia et al., 2018 [8]	Four contacts along the striatal axis (nucleus accumbens to caudate)	7	Baseline, and after every stimulation period (3 months each). Baseline, contact 0, and best contact are reported	Free Cues Serial Recall Test, Stroop Color-Word interference Test, Phonological and Categorical verbal fluency, Letter-number sequencing (WAIS-IV), Trail Making Test (TMT) A and B, Wisconsin Card Sorting Test	No neuropsychological adverse effects were recorded. Statistically significant effects were observed for TMT-B performance times. Performance times at Best Contact (BC) were not significantly different from baseline
Tyagi et al., 2019 [44]	Anteromedial subthalamic nucleus (amSTN) and ventral capsule	6	Baseline, 3, 6, 9, 12, 15 months post-op	Cambridge Neuropsychological Test Automated Battery (CANTAB) and Intra-Extra Dimentional Set (EDS)-shift task	Stimulation of amSTN resulted in improvement of EDS errors, indicating improvement of cognitive flexibility
Parvaresh- Rizi et al., 2022 [37]	Internal capsule and nucleus accumbens (NAc)	4	Baseline, 4, 8, and 12 months post-op	Wisconsin Card Sorting Test (WCST) and Wechsler Memory Scale-Fourth Edition (WMS-IV)	No substantial cognitive impairments. Cognitive improvements were related to the severity of OCD, and two patients with significant improvements in OCD symptoms showed improvement in WCST and WMS-IV

One common concern among patients with OCD, and a possible barrier for undergoing DBS, is the perceived risk for stimulation-induced side-effects often specified as unwanted changes of the mind or personality [36]. In our patients, there was a slight decline in the “learning” subtest of the BVMT-R. However, several of the patients who showed reduced learning also showed improved short-term memory results (although these did not reach statistical significance at a group level). Improvement regarding visual memory and working memory in patients with OCD after DBS has also been noted in other studies [1, 7, 24]. We found a significant improvement in the inhibition/switching subtest of the D-KEFS Color-Word Interference task, which requires switching between two response sets, inhibitory control and word reading, i.e., cognitive flexibility. This finding is consistent with previous reports of improved cognitive flexibility following DBS in patients with OCD [7, 29, 47]. One blinded study comparing (in the same patients) DBS in the anteromedial subthalamic nucleus (amSTN) versus DBS in ventral capsule/ventral striatal (VC/VS) showed improvements in cognitive flexibility after stimulation in the STN, but not in the VC/VS [47]. This finding would suggest improvement specific to DBS in the STN target in these patients. It has been proposed that such cognitive improvement might be an epiphenomenon and that the overflow of obsessive thoughts causes an overload on the executive system and thus also cognitive impairment [3]. This model suggests that the excess of obsessive thoughts in OCD is associated with hyperactivity of the frontostriatal system. DBS in various nodes along the circuitry of OCD would therefore inhibit or decrease this pathological hyperactivity and contribute to a “release” of the executive system.

Apart from the studies presented in Table 5, one meta-analysis of DBS in psychiatric disorders, including eight studies focusing on OCD, concluded that surgery did not result in any cognitive changes [10]. Since methodological limitations sometimes result in small effect sizes, subtler cognitive changes might go undetected. A more recent systematic review of randomized controlled trials [50] and a systematic review on cognitive outcome after DBS for refractory OCD [49] concluded that there is little evidence of adverse effects of DBS. On the other hand, the evidence does not support a positive impact of DBS on cognitive functioning either. Since most of the studies included in these systematic reviews used different instruments at different time-points, it would be difficult to pool the data to provide reliable comparisons.

As early as 2010, one multi-center study [23] described how the implantation site systematically became more posterior (and thus inferior, closer to the caudal nucleus accumbens) during the course of the study program, in order to achieve better outcome of stimulation. More recently, a similar finding has been presented, identifying a set of functionally connected brain regions associated with optimal outcome [28]. Connectivity to the anterior cingulate cortex, insula, and precuneus was predictive of a favorable outcome, regardless of target choice. It has also been suggested that several of the targets used in OCD-DBS are part of a network whose engagement is associated with symptom improvement [20]. The same authors conclude that the most efficacious DBS target region is the anterior hypothalamus in the vicinity of the ITP (inferior thalamic peduncle) and BNST. They also note how closely related the BNST is to amygdalar function and highlight the evidence of the BNST DBS being superior to stimulation of the nucleus accumbens for alleviation of OCD symptoms.

One study including nine participants with severe treatment-refractory OCD [35] demonstrated that DBS of the BNST region substantially alleviated symptoms, with a mean Y-BOCS reduction of 49.6%. When comparing ALIC stimulation to BNST, Luyten et al. [29] found that when they restricted the analysis to patients receiving stimulation in the BNST, 67% were identified as responders during the initial phase of the trial and at last follow-up, that number increased to 83%. As mentioned earlier this is, to our knowledge, the only previous study evaluating BNST-DBS in patients with refractory OCD which also included cognitive data. The findings indicated that DBS of the BNST led to improved executive functioning, increased processing speed, mental flexibility, verbal learning, and memory [29].

The BNST has classically been associated with sustained anxiety responses [5, 44, 52] and considered part of the extended amygdala due to its location and strong structural and functional interactions with this medial temporal lobe structure [14]. Both the amygdala and the BNST seem to be engaged when an individual is faced with an actual threat. However, the BNST is also activated during imagery of a future threat [15, 45]. The BNST thereby mediates the anticipatory phase of a threat and the general apprehension due to the threat, and loss of control to prevent a threat. When the actual physical threat is present, both the BNST and the amygdala will be activated [27]. It has therefore been suggested that the neurobiological basis of hyper-vigilant threat monitoring may be more BNST-dependent and less dependent on the amygdala [44]. This model makes the BNST interesting concerning the etiology of stress-related psychiatric disorders generating anxiety, and hence a promising target for DBS in patients with refractory OCD.

Even in our patients that had a partial response to DBS, the data regarding the impact of DBS surgery on their life showed a marked improvement in terms of independent living and resumption of social interaction. This may be interpreted as a consequence of a reduction in anxiety and better ability to cope with residual symptoms of OCD. One important addition to the growing body of evidence supporting DBS in the BNST area in severe cases of OCD is the additive effect of CBT in combination with DBS, which was discussed in a recently published paper [51]. This is an interesting and important aspect of future research in this field.

## Limitations

With only 8 participants, our sample is relatively small, a fact that we share with several of the few cognitive studies on DBS for OCD (Table 5). Additionally, there was no control group and our design was non-blinded which raises the possibility of potential practice effects or clinical bias. However, the results from this 12-month follow-up of DBS in BNST are in line with a previous study of cognitive effects of DBS in the same target [29], in which patients were assessed shortly after surgery (3–6 months). This finding indicates that the cognitive effects may also be valid up to 12 months after surgery.

## Conclusions

DBS in the area of BNST in patients with treatment-refractory OCD generated very few cognitive effects: on the one hand, a decline on a test of visuospatial learning and, on the other hand, an improvement in cognitive flexibility. Hence, the results of the present study suggest that BNST could be a cognitively safe target for DBS in patients with severe OCD.
